# The Sandwell Project: A controlled evaluation of a programme of targeted screening for prevention of cardiovascular disease in primary care

**DOI:** 10.1186/1471-2458-8-73

**Published:** 2008-02-25

**Authors:** Tom Marshall, Paul Westerby, Jenny Chen, Mary Fairfield, Jenny Harding, Ruth Westerby, Rajai Ahmad, John Middleton

**Affiliations:** 1 Department of Public Health & Epidemiology, University of Birmingham, Edgbaston, Birmingham, B15 2TT, UK; 2 Public Health Department, Sandwell Primary Care Trust, Kingston House, 438 High Street, West Bromwich, West Midlands, B70 9LD, UK; 3 Cardiology Department, Sandwell District Hospital, Lyndon, West Bromwich, West Midlands B71 4HJ, UK

## Abstract

**Background:**

A pilot cardiovascular disease prevention project was implemented in the inner-city West Midlands. It was evaluated by comparing its effectiveness to a control group where full implementation was delayed by a year.

**Methods:**

Cardiovascular risk factor data were extracted on all untreated patients 35 to 74 years old from electronic medical databases in six general practices. A best estimate of ten-year CVD risk cardiovascular risk was calculated on all patients using the extracted risk factor data. Default risk-factor values were used for all missing risk factor data. High risk patients were thus identified. In four practices a project nurse systematically invited, assessed and referred high risk patients for treatment. Two control practices were provided with a list of their high risk patients. The outcomes were the proportions of untreated high-risk patients who were assessed, identified as eligible for treatment and treated under two strategies for identifying and treating such patients in primary care.

**Results:**

Of all high-risk patients suitable for inclusion in the project, 40.6% (95% CI: 36.7 to 45.7%) of patients in intervention practices were started on treatment were started on at least one treatment, compared to 12.7% (95% CI: 9.8% to 16.1%) in control practices.

**Conclusion:**

A strategy using electronic primary care records to identify high risk patients for CVD prevention works best with a process for acting on information, ensuring patients are invited, assessed and treated.

## Background

Current clinical guidelines largely use individuals' estimated ten-year risk of developing cardiovascular disease (CVD) to determine eligibility for treatment [1]. Risk of CVD is derived using the Framingham CVD risk equation [2]. This requires data on the patient's age, gender, diabetic status, whether they have CVD, smoking status, blood pressure, total and HDL cholesterol levels. Any individual whose risk of developing CVD within the next 10 years exceeds 20% is eligible for treatment.

Patients with existing CVD are the highest priority in any prevention programme. In the UK, general practices are responsible for the primary health care needs of a registered population of patients. General practices are offered incentives to identify and treat these patients in the Quality and Outcomes Framework of the Department of Health [3]. Prevention in those without CVD but at high risk of developing CVD was a priority in the National Service Framework for Coronary Heart Disease, but is not specifically covered under current incentives [4]. This pilot therefore focused only on CVD primary prevention.

CVD prevention in those without existing CVD requires identification of patients eligible for treatment and subsequent treatment. Usual current practice is to assess patients' risk factors opportunistically. In many cases risk factor data (such as age, sex, diabetic status, smoking history and blood pressure) are already recorded in practice databases. This information could be used to prioritise patients for CVD risk factor assessment [5].

Sandwell is a deprived urban area in the West Midlands of England, 20.3% of the population are from minority ethnic groups (14.0% South Asian and 3.8% Afro-Caribbean) [6]. From 2003 to 2005 the standardised mortality rates for coronary heart disease were 138 in males and 165 in females under 75 and for stroke 177 and 156 [7].

This paper reports the evaluation of a pilot CVD prevention project in Sandwell. The project was implemented in a stepped manner (in effect as a controlled trial) in order to evaluate the effectiveness of the project. In the intervention group, patients without existing CVD and not currently on antihypertensive treatment, were identified from electronic primary care records. From these untreated patients were identified those patients likely to be at high risk of CVD. High risk patients were invited for CVD risk factor assessment by a project nurse, assessed by the project nurse and if they were found to be eligible for treatment they were referred to the GP for prescription of drug treatments. In the control group, full implementation was delayed for a year. At the start of the year untreated patients likely to be at high risk of CVD were identified in a similar manner to the intervention group. The practices responsible for these patients were provided with information identifying these patients as likely to be at high-risk but made their own arrangements for assessment and treatment. It was believed that the project would increase the proportion of high risk patients assessed and therefore increase the proportion of eligible patients started on treatment. The intervention (systematic invitation by the project nurse) was extended to the control group one year after the start of the project.

## Methods

### Population under study

The primary care trust identified general practices in Sandwell Primary Care Trust (PCT) located in areas of the PCT with the highest standardised mortality rates for CVD. To facilitate data extraction, only practices that used Torex electronic medical records databases were included. Participating practices agreed to assess the patients identified by the search strategy as probably at high risk and to follow the local protocol for prescribing preventive treatments. (Table 1) Six practices took part in the project. Four smaller practices were selected as the intervention group and two larger practices were selected as the control group. The intervention group practices consisted of three single partner practices and a nurse practitioner partnership; the control group practices each had two partners. The project was registered for research governance with Sandwell Primary Care Trust, who determined that since this was an evaluation of organisational change aimed to improve access to freely available health services, formal ethics committee approval was not required.

**Table 1:** Sandwell Primary Care Trust's local protocol for prevention of CVD in primary care

**Intervention**	**Treatment eligibility criteria**
Aspirin 75 mg	Age ≥50 and calculated risk ≥20%

Antihypertensive treatment:	Blood pressure ≥160/100 mm Hg
1. Bendroflumethiazide 2.5 mg	
2. Lisinopril 5 mg	Blood pressure ≥140/90 mm Hg calculated ten-year CVD risk ≥20%
3. Bisoprolol 10 mg	

Simvastatin 40 mg	Total cholesterol ≥7 mmol/l
	Diabetic: age ≥40
	Diabetic: total cholesterol ≥6 mmol/l
	Any cholesterol level calculated ten-year CVD risk ≥20%

### Data extraction

Patients were eligible for inclusion in the prevention project if they were aged 35 to 74, did not have an existing diagnosis of CVD and were not currently receiving a prescription for antihypertensive treatment. A software query compatible with Torex databases was written to identify these patients and to extract their CVD risk factor data. The extracted data included age, sex, diabetic status, smoking status, the most recent three blood pressures extracted within the last three years, the most recent total cholesterol and HDL cholesterol levels within the last three years and the practice's own patient identifier (a patient number known only to the practice). Five practices were visited and had data extracted in July 2005 and in one practice in February 2006.

### Determining risk and prioritising patients for assessment

In each of the six practices, extracted CVD risk factor data were downloaded into an Excel spreadsheet and an estimated CVD risk was calculated for all the included patients using the Framingham risk equation [8]. Where more than one blood pressure or cholesterol measurement was available, the average of all the available measurements was used to calculate risk. Where a risk factor was not known, a default (or prior estimate) of the risk factor status was used instead. In other words, CVD risk was calculated using whatever risk factor information was available on patients. In most cases this information was incomplete because of missing cholesterol levels, blood pressures or smoking status. This approach follows a previously described method [9,10].

The spreadsheet of CVD risks was returned to the practice and then matched to the practice patients. In the Excel spreadsheet patients were ranked from top to bottom in descending order of CVD risk. In all six practices, probable high-risk patients (patients whose ten-year CVD risk exceeded 20%) were identified, the list was explained to practice staff and the names were checked by practice staff to exclude patients who had died, had illnesses that made them unsuitable for CVD risk screening (e.g. terminally ill) or had moved away.

### Identification, assessment and treatment: intervention group

In the four intervention practices the project nurse undertook a strategy of active invitation and assessment of patients. Patients identified as probably at high-risk of CVD were mailed an appointment to attend a CVD assessment clinic in their practice. Patients who did not either attend or reschedule their appointment were sent a second and a third letter or telephoned (if a telephone number was available). When patients attended, the project nurse determined ethnicity, assessed lifestyle risk factors including smoking history, drinking habit, waist and hip measurement and ratio, weight and height and body mass index; family history of CVD and measured blood pressure and pulse. A fasting blood test was arranged including renal and liver function tests, fasting lipids and fasting glucose. The project nurse calculated CVD risk, determined eligibility for drug treatments and if appropriate discussed the potential benefits of treatment using a risk and benefit calculator [11]. Eligible patients were referred to the GP for prescription of drug treatments. Appropriate patients were referred to smoking cessation services, for exercise on prescription or for dietary advice. Outcomes were assessed one year after the intervention began. Patients

### Identification, assessment and treatment: control group

In the two control practices, the practice took responsibility for arranging assessment and treatment of probable high-risk patients during the year of the study. Neither control practice systematically invited patients, both preferring to assess patient opportunistically when they attended for other reasons. One year after the project began the project nurse was made available to the control group practices.

### Evaluation

One year after CVD risk factor data were first extracted, a second data extract was undertaken to assess outcomes. Patients were considered to have been assessed if there was a record of cardiovascular risk factor status (blood pressure and cholesterol levels) in their electronic medical records. They were considered to be eligible for treatment if their recorded risk factors indicated that they met the criteria in Table 1. The primary outcome was the number of eligible patients who were started on treatment from the list of those identified as probably at high risk of CVD. Secondary outcomes were the numbers of probable high-risk patients assessed; the numbers identified as eligible for treatment; the numbers of eligible patients started on treatment. We also report the numbers of patients referred to smoking cessation services, for exercise on prescription or dietary advice. Statistical analysis was carried out using EpiInfo 6.0.

## Results

**Figure 1:**
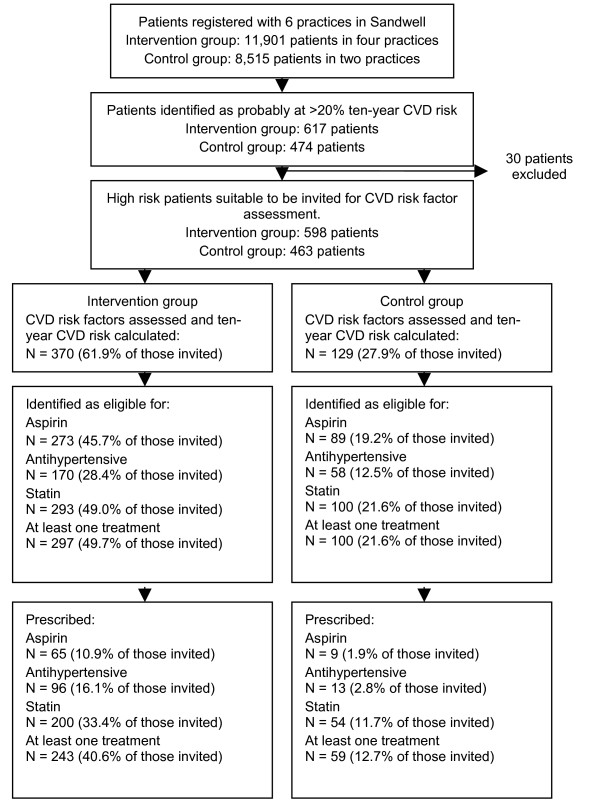
Flow chart of the project evaluation.

The six practices included 1091 patients identified as aged 35 to 74, without a diagnosis of CVD, not taking antihypertensive treatment, but probably at high risk. Of these patients 30 (2.7%) were excluded because they were unsuitable to be invited for CVD risk assessment: 25 were not on the practice list and five were in hospital for more than six months (Figure 1 and Table 2).

**Table 2:** Numbers of patients aged 35 to 74, free from CVD and not taking antihypertensive drugs and numbers whose ten-year CVD risk* probably exceeds 20%

Practice	Practice list	Number aged 35–74, free from CVD and not taking antihypertensive drugs (percentage of practice list)	Number with ten-year CVD risk* probably >20% (percentage of practice list)
PN1	2,029	681 (33.6%)	106 (5.2%)
PN2	2,825	894 (31.6%)	129 (4.6%)
PN3	3,447	1,192 (34.6%)	177 (5.1%)
PN4	3,600	1,149 (31.9%)	205 (5.7%)
All project nurse practices	11,901	3,916 (32.9%)	617 (5.2%)

Info1	4,005	1,371 (34.2%)	182 (4.5%)
Info2	4,510	1,626 (36.1%)	292 (6.5%)
All information only practices	8,515	2,997 (35.2%)	474 (5.6%)

All practices	20,416	6,913 (33.9%)	1,091 (5.3%)

* See text for an explanation of the way in which ten-year CVD risk is calculated

### Suitable patients assessed

Of the 1061 patients suitable for CVD risk assessment, 499 (47.0%) were fully assessed during the project, 61.9% in the intervention group and 27.9% in the control group. Compared to information only practices, the relative risk of being assessed was 2.22 (95% CI: 1.89 to 2.60) in intervention group practices (Table 3).

**Table 3:** Numbers of patients eligible for CVD risk assessment, numbers assessed and numbers identified as eligible for drug treatments

Practice	Eligible for CVD risk assessment*	CVD risk calculated (% of those eligible for assessment)	Identified as eligible for aspirin (% of those eligible for assessment)	Identified as eligible for antihypertensive (% of those eligible for assessment)	Identified as eligible for statin (% of those eligible for assessment)	Identified as eligible for at least one treatment (% of those eligible for assessment)
PN1	103	60 (58.3%)	40 (38.8%)	22 (21.4%)	43 (41.7%)	44 (42.7%)
PN2	128	m (69.5%)	65 (50.8%)	29 (22.7%)	69 (53.9%)	70 (54.7%)
PN3	164	88 (53.7%)	70 (42.7%)	52 (31.7%)	76 (46.3%)	m (46.3%)
PN4	203	133 (65.5%)	98 (48.3%)	m (33.0%)	105 (51.7%)	107 (52.7%)
All project nurse practices	598	m (61.9%)	273 (45.7%)	170 (28.4%)	293 (49.0%)	297 (49.7%)

Info1	175	75 (42.9%)	51 (29.1%)	36 (20.6%)	57 (32.6%)	57 (32.6%)
Info2	288	54 (18.8%)	38 (13.2%)	22 (7.6%)	43 (14.9%)	43 (14.9%)
All information only practices	463	129 (27.9%)	89 (19.2%)	58 (12.5%)	100 (21.6%)	100 (21.6%)

All practices	1061	499 (47.0%)	362 (34.1%)	228 (21.5%)	393 (37.0%)	397 (37.4%)

* In project nurse practices these patients were actively invited. In the information only practices they were seen opportunistically when they consulted for other reasons.

### Suitable patients identified as eligible for treatment

In total 397 (37.4%; 95% CI: 34.5% to 40.4%) of patients suitable for CVD risk assessment were found to be eligible for at least one drug treatment. Compared to information only practices, the relative risk of being found to be eligible for drug treatment in the project nurse practices was 2.30 (95% CI: 1.90 to 2.78).

In the intervention practices more patients the following proportions of patients eligible for inclusion were identified as eligible (49.7%) for any preventive treatment, (45.7%) for aspirin, (28.4%) for antihypertensives and (49.0%) for statins. In the control practices the equivalent proportions identified as eligible for treatment were (21.6%) for any preventive treatment, (19.2%) for aspirin, (12.5%) for antihypertensives and (21.6%) (Table 3).

### Probable high-risk patients suitable for CVD risk assessment who were started on treatment

In the intervention practices, 40.6% of all high-risk patients suitable for inclusion in the project were started on at least one treatment, compared to 12.7% in the information only practices. This is a relative risk of 3.19 (95% CI: 2.47 to 4.12).

### Proportions of probable high-risk patients referred to other services

In the intervention practices more patients were referred to Sandwell Physical Activity Referral Scheme for advice on physical activity (6.4% v 0.9%; P < 0.001 by Chi squared test) and for dietary advice on weight loss (1.5% v 0.0%; P = 0.006 by Fishers exact test). The proportions of smokers were referred to smoking cessation services were not significantly different (18.8% v 15.3%; P < 0.536 by Chi squared test) (Table 4).

**Table 4:** Numbers of patients referred to other services

Practice	Referred for advice on physical activity (%)*	Referred to dietician (%)*	Referred to smoking cessation service (% of smokers)
PN1	9 (8.7%)	2 (1.9%)	7 (6.8%)
PN2	10 (7.8%)	1 (0.8%)	13 (10.2%)
PN3	14 (8.5%)	3 (1.8%)	4 (2.4%)
PN4	7 (3.4%)	4 (2.0%)	18 (8.9%)
All project nurse practices	40 (6.7%)	10 (1.7%)	42 (7.0%)

Info1	3 (1.7%)	0 (0.0%)	10 (5.7%)
Info2	1 (0.3%)	0 (0.0%)	1 (0.3%)
All information only practices	4 (0.9%)	0 (0.0%)	11 (2.4%)

All practices	44 (4.1%)	10 (0.9%)	53 (5.0%)

* Percentage of all those suitable for assessment (see Table 3 for denominator)

### Eligible patients started on treatment

Further analysis was carried out in order to determine whether eligible patients were equally likely to be started on treatment in intervention and control practices. In total 302 patients found to be eligible for drug treatment were started on at least one appropriate treatment: 81.8% of those eligible in the intervention group and 59.0% of those eligible in the control group. Compared to information only practices, the relative risk of starting an eligible patient on at least one appropriate treatment in the project nurse practices was 1.39 (95% CI: 1.17 to 1.65).

In intervention practices eligible patients were significantly more likely to be started on aspirin (23.8% v 10.1%. Relative risk 2.35; 95% CI: 1.22 to 4.53), antihypertensives (56.5%; 22.4%. Relative risk 2.52; 95% CI: 1.43 to 4.14) and statins (68.3% v 54.0%. Relative risk 1.26; 95% CI: 1.04 to 1.54) (Table 5).

**Table 5:** Numbers of patients eligible for and started on aspirin, antihypertensives and statins

	Aspirin		Antihypertensives		Statin		Any Rx	
Practice	Eligible	On treatment (% of eligible)	Eligible	On treatment (% of eligible)	Eligible	On treatment (% of eligible)	Eligible	On treatment (% of eligible)
PN1	40	10 (25.0%)	22	13 (59.1%)	43	23 (53.5%)	44	33 (75.0%)
PN2	65	11 (16.9%)	29	19 (65.5%)	69	59 (85.5%)	70	62 (88.6%)
PN3	70	14 (20.0%)	52	27 (51.9%)	76	56 (73.7%)	76	65 (85.5%)
PN4	98	30 (30.6%)	67	37 (55.2%)	105	62 (59.0%)	107	83 (77.6%)
All project nurse practices	273	65 (23.8%)	170	96 (56.5%)	293	200 (68.3%)	297	243 (81.8%)

Info1	51	3 (5.9%)	36	9 (25.0%)	57	31 (54.4%)	57	35 (61.4%)
Info2	38	6 (15.8%)	22	4 (18.2%)	43	23 (53.5%)	43	24 (55.8%)
All information only practices	89	9 (10.1%)	58	13 (22.4%)	100	54 (54.0%)	100	59 (59.0%)

All practices	362	74 (20.4%)	228	109 (47.8%)	393	254 (64.6%)	397	302 (7 6.1%)

* Percentage of all those suitable for assessment (see Table 3 for denominator)

## Discussion

### Main finding of this study

Providing practices with information identifying patients who were probably at greater than 20% ten-year CVD risk resulted in significant numbers of new patients eligible for treatment being identified and started on treatment. We found that the presence of a project nurse in the practices more than doubled the probability of patients being assessed and hence more than doubled the number of patients identified as eligible for treatment. More surprisingly, among patients identified as eligible for treatment, the presence of a project nurse significantly increased the probability that eligible patients were prescribed preventive treatments. Overall, the project nurse therefore trebled the chances of being identified as eligible for and subsequently started on a preventive treatment. There were also greater numbers of referrals for advice on physical activity or diet.

There is some further evidence that the opportunistic strategy does not always result in patients having their risk factors measured. In the control group practices 73.0% (95% CI: 62.6% to 81.9%) of patients who saw the GP twice or more had all their risk factors assessed. By contrast, in the intervention practices, all patients who saw the project nurse twice or more had all their CVD risk factors assessed.

There is also some further evidence that general practitioners do not always start treatment in patients whose risk factors are known and who are known to be eligible for treatment. Among the 1091 untreated patients initially selected for inclusion in the study, 116 (10.6%: range 1.9% to 30.8% in the six practices) had sufficient risk factor information to calculate CVD risk (a smoking history, two blood pressures and a total cholesterol level) already in their electronic medical records. Hence it was already possible to determine that they were eligible for treatment before the project began.

The invitation strategy was clearly superior to opportunistic assessment: a quarter of patients saw their GPs twice or more but did not have their risk factors assessed. Nevertheless, even with systematic invitation, one third of patients did not attend. Few patients who were offered treatment declined it. The weakest link in the process was GP prescribing. Even with the project nurse present, GPs did not prescribe antihypertensives to half of eligible patients or aspirin to four fifths. While the presence of a project nurse appeared to improve both identification and prescribing, there is clearly scope for further improvement.

### What is already known on this topic?

There are studies of other interventions to improve identification of patients for CVD prevention in primary care. Over time, continuous feedback may improve identification of patients with CVD [12]. Changes made to primary care electronic medical records have successfully increased rates of CVD risk factor assessment [13]. In Scotland the CARDIA system can identify untreated patients eligible for CVD prevention and prompts GPs to assess risk factors and prescribe [14]. However the effectiveness of this system on prescribing has not been evaluated.

Electronic reminders have been used in a number of settings to try to change clinician behaviour within the existing health care system. However results in clinical practice have been mixed. In a US outpatient clinic setting, electronic reminders had no effect on the management of heart disease [15]. Among Italian general practitioners, a reminder increased prescribing of antiplatelet therapy for patients at high risk of CVD [16]. Electronic reminders have been demonstrated to increase vaccination levels [17-19].

A more sophisticated intervention has extracted data from general practices and fed this back to practices to help them target hypertension management [20]. This achieved some success. Evidence suggests that the effects of electronic reminders on clinician behaviour are dependent on organisational factors and other incentives [21-23].

Some studies report increases in prescribing of preventive drugs. Searches of electronic medical records have been used to identify patients with diabetes and increase the proportions in whom aspirin was prescribed [24]. Complex interventions involving guidelines, audit, prompts and practitioner education have succeeded in improving prescribing practice for hypertension [25]. A similar approach has improved identification and treatment of atrial fibrillation [26].

### What this study adds

Information alone is insufficient to ensure action is taken. Indeed, in one (information only) practice nearly one third of untreated high risk patients already had sufficient risk factor information in electronic medical records to identify them as eligible for treatment. A prevention strategy is most likely to succeed if a system is in place to ensure that information is acted upon.

The information only practices in the Sandwell Cardiovascular Prevention Project received an intervention similar to previous studies: information was provided to try to change clinician behaviour within the existing primary health care system. However the project nurse practices received a somewhat different intervention.

Compared to other approaches to primary prevention the strategy implemented in these practices was relatively simple. Make use of information technology to identify patients. Ensure that it is someone's specific responsibility to invite identified patients, assess them, determine their eligibility for treatment and discuss their treatment preferences. Then present this information to the prescriber. Effectively this creates a new system for CVD prevention. Rather than expecting the existing primary care system to act on information, a separate service was created to respond to the information. This reflects the experience of industry that it is not the presence of information technology but the way in which it is used, that is critical to its success [27]. It is of note that despite identifying patients, assessing them and providing this information to general practitioners, many patients were not prescribed treatments for which they were eligible. This meant that within this strategy the greatest deviation from optimum practice was prescribing. This was also the part of the strategy which most relied on changing behaviour within the existing system. There may be wider lessons for making use of patient data and information technology to improve to patient care.

## Conclusion

There are difficulties interpreting this evaluation. There may be systematic differences between the practices other than the presence of the project nurse. However, all four project nurse practices were consistently better than both control practices. Aspirin use may be under recorded because it can be obtained over the counter. Nevertheless there was clearly higher aspirin use in project nurse practices. The identification and project nurse strategy may not generalise to other settings with larger multi-partner practices. This last question can only be addressed with further research.

## Competing interests

The author(s) declare that they have no competing interests.

## Authors' contributions

TM designed the prevention strategy & evaluation. It was implemented and overseen by PW, MF, AR, JH, JC and TM. Data was collected by PW. All authors contributed to writing the paper.

## Pre-publication history

The pre-publication history for this paper can be accessed here:


